# Methotrexate-associated reversible liver atrophy in a patient with rheumatoid arthritis

**DOI:** 10.1093/rap/rkaa014

**Published:** 2020-05-06

**Authors:** Tomohiro Sugimoto, Sho Mokuda, Eiji Sugiyama

**Affiliations:** r1 >Department of Internal Medicine, Miyoshi Central Hospital, Miyoshi; r2 Department of Clinical Immunology and Rheumatology, Hiroshima University Hospital, Hiroshima, Japan

**Fig. 1:**
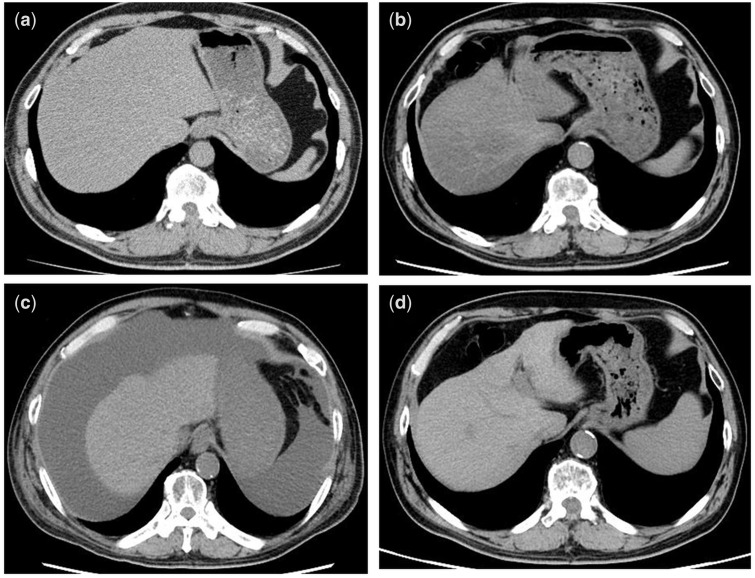
CT scan of a patient with MTX-associated reversible liver atrophy in a patient with RA (**a**) Seven years before hospitalization. (**b**) Three years before hospitalization. (**c**) At the time of hospitalization. (**d**) Ten months after MTX discontinuation.

A 62-year-old man with RA for ≥10 years who presented with abdominal bloating was hospitalized. He was receiving MTX (10 mg/week), iguratimod and bucillamine, and he showed no evidence of viral hepatitis, diabetes mellitus or alcoholism. His aspartate aminotransferase (AST) and alanine aminotransferase (ALT) levels were 79 and 53 U/l, respectively. His platelet count was 50×10^3^/µl. The fibrosis 4 (FIB-4) score, {[Age (year) x AST (U/l)] / (platelet (x10^3^/µl) x √ALT (U/l))}, was 13.46 (advanced liver fibrosis). CT performed every few years before hospitalization showed gradually progressing liver atrophy (Fig. 1a and b: 7 and 3 years before hospitalization, respectively). CT performed at the time of hospitalization showed severe liver atrophy and ascites (Fig. 1c). Then, MTX treatment was discontinuated. Ten months after drug discontinuation, his AST and ALT levels reduced to 25 and 20 U/l, respectively, and the platelet count and FIB-4 score were 109×10^3^/µl and 3.23 (moderate liver fibrosis), respectively. The ascites disappeared, and the liver atrophy slowly improved (Fig. 1d).

Among all types of MTX-induced liver damage, liver cirrhosis is rare [1]. To our knowledge, there are no reports of radiological changes in the liver and ascites in RA patients receiving MTX [2]. Weekly MTX might induce reversible chronic hepatotoxicity; therefore, in cases of liver atrophy, physicians should discontinue MTX and monitor patients using CT.

*Funding*: No specific funding was received from any funding bodies in the public, commercial or not-for-profit sectors to carry out the work described in this manuscript.

*Disclosure statement* **:** The authors have declared no conflicts of interest.
